# One filter may be enough for duplicate inferior vena cava filter implantation in patients with deep venous thrombosis: Two cases report

**DOI:** 10.1097/MD.0000000000032480

**Published:** 2022-12-30

**Authors:** Tao Li, Qi Wang, Wei Wang, Jun Yang, Shuilin Dong

**Affiliations:** a Division of Vascular Surgery, Hepatic Surgery Center, Tongji Hospital, Tongji Medical College, Huazhong University of Science and Technology, Wuhan, China.

**Keywords:** deep venous thrombosis, duplicate inferior vena cava, inferior vena cava filter

## Abstract

Duplicate inferior vena cava (IVC) is an uncommon congenital malformation, but should be treated carefully under the circumstances of deep venous thrombosis (DVT). However, there is a paucity of clinical guidance on this situation.

**Methods:**

Duplicate IVC was diagnosed based on the imaging examination that revealed the dual IVC. Deep venous thrombosis was diagnosed by Compression Doppler ultrasonography of both lower extremities with the high-elevated D-dimer. Retrievable IVC filters were implanted to prevent massive and fatal pulmonary embolism. Appropriate anticoagulation therapy was also performed.

**Results:**

Two retrievable filters were successfully implanted and retrieved in two patients with deep venous thrombosis and duplicate inferior vena cava, respectively. During further follow-up, no adverse event was reported.

**Conclusion:**

Comprehensive imaging examination might contribute to the diagnosis of duplicate IVC, especially when individual conditions were limited. The position above the confluence of bilateral IVCs might be an appropriate suprarenal retrievable filter insertion location. To deal with different types of dual IVC anatomy, different strategies should be taken into consideration.

## 1. Introduction

Congenital anomalies of the inferior vena cava (IVC) originate from dysplasia of the venous system in the early stage of embryogenesis.^[1]^ Furthermore, the failure regression of the left supra cardinal vein might result in the formation of duplication of IVC.^[2]^ Previous research reported a low prevalence of doubled IVC from 0.2% to 3%.^[3–9]^ IVC anomalies present mostly nonspecific or no symptoms and are often discovered accidentally by autopsy or imaging examination.^[10]^ However, it is important to recognize and report such malformation to reduce related complications during abdominal operations, such as IVC filter placement.^[7,8]^ Even if an IVC filter has been inserted, there is still a risk of development of pulmonary embolism (PE), owing to double IVC providing extra access for thrombus from the lower extremities.^[3,11]^ Thus, when patients develop venous thromboembolism with the diagnosis of duplicated IVC, it is essential to carefully consider the position, size and number of the IVC filter in order to effectively prevent massive and fatal PE. Hereby, we have presented 2 cases and introduced our strategies for filter insertion in patients with dual IVC. Both 2 patients provided informed consent for publication of the case.

## 2. Case presentation

### 2.1. Case 1

A 29-year-old woman was admitted to our hospital due to pain and edema of the left lower limb for 6 days. 12 days ago, she accepted in vitro fertilization-embryo transfer and elevated serum levels of human chorionic gonadotropin indicated her pregnancy state. She had no medical history before and known allergies. On admission, laboratory and imageological examination were performed. A high concentration (21μg/mL) of serum D-dimer was reported. Protein C and antithrombin were below the normal limits, which might reveal thrombophilia as part of the etiology of thrombosis. Compression Doppler ultrasonography of both lower extremities demonstrated extensive deep venous thrombosis (DVT) in the left extremity involving the iliac, femoral, popliteal, calf, and great saphenous vein. Furthermore, computed tomography pulmonary angiography showed bilateral PE and a small amount of pleural effusion.

**Figure 1.**
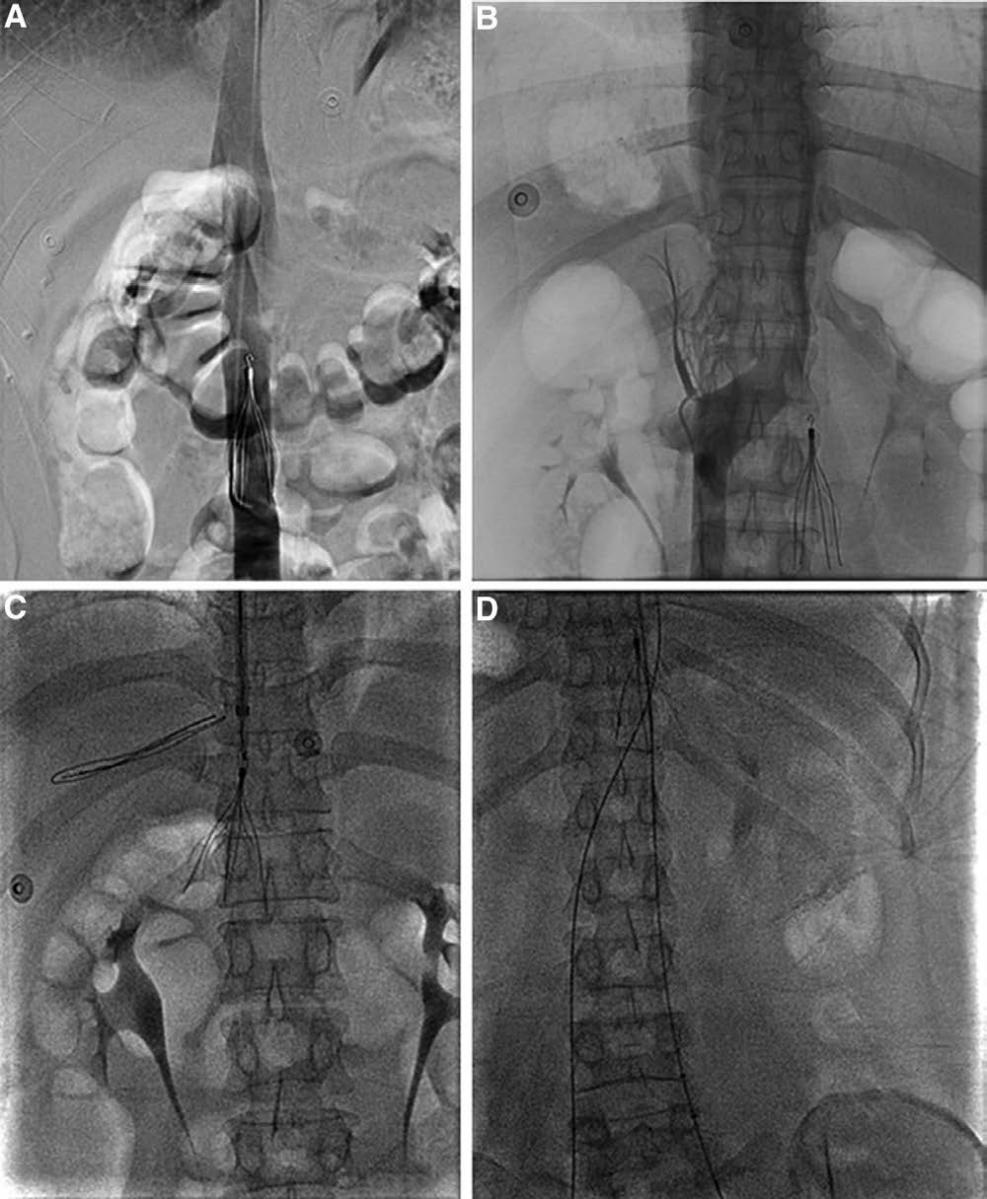
Venography: (A) The filter was placed below the ostium of left IVC, which was mistaken for the left renal vein; (B) Left IVC was demonstrated before mechanical thrombectomy; (C) The filter was readjusted to the position above the confluence of bilateral IVC; (D) Percutaneous mechanical thrombectomy through AngioJet system.

After she was informed of her medical dilemma of early pregnancy and the high risk of thrombus progression and fatal PE, she and her relatives decided to terminate the pregnancy and receive interventional treatment. Then IVC filter implanting and percutaneous mechanical thrombectomy through AngioJet system (Boston Scientific) was planned. After topical anesthesia, her right femoral vein was punctured using Seldinger puncture technique. Before filter insertion, angiography was performed to detect anatomic characteristics and patency of IVC. Then an infrarenal Celect retrievable filter (Cook) was inserted (Fig. 1A). However, a duplicate IVC was demonstrated during venography before mechanical thrombectomy (Fig. 1B). Nevertheless, the left IVC was not wide enough for fully stretch of normal filters and insertion of another filter was not feasible. Thus, we selected the suprarenal position above the confluence of the left and right IVC as new filter insertion site. The filter was dragged into the recovery sheath and then released in the planned position through jugular approach (Fig. 1C and D). The following mechanical thrombectomy successfully removed most of thrombus in deep vein of left lower limb and her symptoms were relieved. After the operation, rivaroxaban was chosen for her further anticoagulation therapy.

One month later, the patient went back to our hospital for filter retrieval. Preoperative examination reported recanalization of main thrombus in deep vein of left lower extremity and no new thrombi formation. The filter was removed safely and successfully. During her 6 months follow-up, no adverse event was reported.

### 2.2. Case 2

A 23-year-old man was investigated by chest enhanced CT scan for 1 month of chest tightness, 2 weeks of cough and shortness of breath without any evident causes. Bilateral and massive PE was reported. Otherwise, dilated pulmonary artery trunk and right atrium might indicate pulmonary hypertension, which was demonstrated by the following doppler echocardiography. Along with the elevated serum D-dimer (2.1 μg/mL), acute extensive DVT in the veins of left and right lower limbs was confirmed by compression doppler ultrasonography. Other laboratory tests were performed to evaluate hypercoagulability and the results suggested a high possibility of antiphospholipid syndrome.

**Figure 2.**
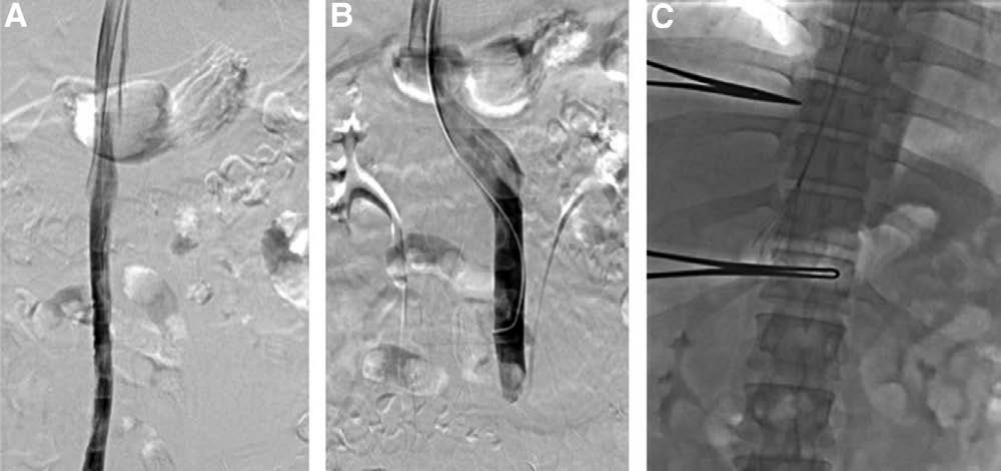
Duplicate IVC: (A) right IVC; (B) left IVC; (C) temporary IVC filter was implanted.

Due to his respiratory symptoms, the patient took electrocardiograph monitoring and low-flow oxygen therapy. To avoid PE aggravating, we planned IVC filter insertion and catheter-directed thrombolysis. After appropriate anesthesia and puncture, catheter was advanced into the IVC through jugular access. Preoperative venography then demonstrated duplicated IVC (Fig. 2A and 2B). Considering DVT happened in both lower extremities, the suprarenal position above confluence of double IVC might be the ideal site for filter placement. Then a Tempofilter (Braun Medical, Germany) was deployed to the aim position (Fig. 2C). Anticoagulant therapy with low molecular weight heparin and warfarin (target INR 2–3) was administrated and no adverse event happened until the patient was discharged from our hospital. His follow-up results showed that thrombus in lower limb vein gradually recanalized and disappeared under the anticoagulation of warfarin. The filter was retrieved successfully 3 months later. Furthermore, no recurrence of thrombus and symptoms was reported during further 6-month follow-up.

## 3. Discussion

IVC anomalies were generally divided into several types: the transposition of IVC with^[12]^ or without^[13]^ regressed right IVC, ipsilateral double IVC,^[14]^ bilateral duplication of IVC and other rare variations, such as marsupial vena cava.^[15]^ However, the duplication of IVC was one of the most common types of these malformations.^[4,16]^

When anticoagulation therapy is contraindicated, IVC filter placement should be considered for preventing PE.^[17]^ In recent years, some studies reported that lower extremity deep vein thrombosis (LEDVT) patients with IVC malformations might benefit from similar interventions conducted on normal LEDVT patients, such as IVC filters implantation, catheter-directed thrombolysis, and thrombus extraction.^[18–20]^ Nicolas et al^[21]^ presumed that multidetector computed tomography angiography and magnetic resonance imaging (MRI) might play a key role in the diagnosis of duplicated IVC. However, other surgeons performed venography^[3]^ and doppler ultrasonography^[10]^ as routine preoperative examinations, in view of limited medical facilities conditions and cost. In our hospital, doppler ultrasound and venography were performed for patients scheduled for filter placement to investigate and evaluate the condition of the whole abdominal venous system. During the venography, the ostia of bilateral renal and iliac vein were generally detected for the optimization of filter position. However, the left iliac vein of the patient in case 1 was embolized by thrombus so that we skipped this step. Thus, we mistook the confluence of bilateral IVC for the ostium of left renal vein. Then doubled IVC was not observed until the second venography before mechanical thrombectomy. Fortunately, we finally readjust the filter to the appropriate position and the patient had no further adverse events. Such fault might suggest the importance of preoperative examination on potential IVC malformations. Furthermore, previous studies showed diagnostic difficulty in different cases by using venography,^[22,23]^ CT or MRI.^[4,24]^ Therefore, integrated investigation approaches might contribute to the diagnosis of duplicated IVC when individual conditions were limited.

The diameters of the vessels and thrombotic condition play key roles in determining appropriate position for IVC filters. Lei Jiang et al^[10]^ reported a case that they only placed one filter in left IVC below the ostium of left renal vein. In this report, LEDVT was only demonstrated in deep vein of left extremity so that one filter in left IVC might be enough to block thrombus effectively. In our 2 cases, we implanted one filter above the confluence of bilateral IVC instead of in one of the IVCs. The diameter of the left IVC in case 1 was under the criteria for complete stretch of filter and in case 2, the filter might be more economical and efficient to prevent thrombus from deep veins of both extremities. However, the renal function should be monitored in case of renal vein thrombosis, because the filters in our cases were also suprarenal. Greenfield^[25]^ et al reported that suprarenal filter placement might receive acceptable clinical results. Their research showed no renal dysfunction in patients with supra- and infrarenal filters. Furthermore, there was no significant difference in recurrent rate of PE and rate of long-term caval occlusion between these 2 groups. The retrieval of the filter might significantly reduce the potential risk of long-term indwelling of filters. In our 2 cases, there was no adverse event reported during follow-up. This might imply that suprarenal filter placed above the confluence of 2 IVCs could be an appropriate and cost-effective choice for treating DVT patients with duplicate IVC. Natsis et al^[26]^ classified the duplicate IVC as 3 types. Type Ⅰ had 2 symmetrical and approximately of the same caliber trunks, which might be suitable for filters setting in each IVC. In recent studies, surgeons implanted filters into both IVCs of the patients and received well clinical outcomes.^[27,28]^ Hence, different types of duplicate IVC anatomy might require different strategies of filter placement. This might need more large-scale studies for further research.

In conclusion, duplicate IVC is rare but of great influence on the treatment of DVT patients. Preoperative imaging examination is very important for assessment of specific IVC anatomy and parameters affecting following filter implantation. Multiple approaches, such as CT, MRI, and venography, should be considered to obtain preferable results. The position above confluence of bilateral IVCs might be an appropriate location for suprarenal retrievable filter placement. Nevertheless, surgeons should take different strategies when facing different types of duplicate IVC for acquiring better clinical prognosis.
